# Phosphodiesterase-4 Inhibition Alters Gene Expression and Improves Isoniazid – Mediated Clearance of *Mycobacterium tuberculosis* in Rabbit Lungs

**DOI:** 10.1371/journal.ppat.1002262

**Published:** 2011-09-15

**Authors:** Selvakumar Subbian, Liana Tsenova, Paul O'Brien, Guibin Yang, Mi-Sun Koo, Blas Peixoto, Dorothy Fallows, Veronique Dartois, George Muller, Gilla Kaplan

**Affiliations:** 1 Laboratory of Mycobacterial Immunity and Pathogenesis, the Public Health Research Institute (PHRI) at the University of Medicine and Dentistry of New Jersey (UMDNJ), Newark, New Jersey, United States of America; 2 Biological Sciences Department, New York City College of Technology, Brooklyn, New York, United States of America; 3 Novartis Institute for Tropical Diseases, Singapore; 4 Celgene Corporation, Summit, New Jersey, United States of America; Harvard School of Public Health, United States of America

## Abstract

Tuberculosis (TB) treatment is hampered by the long duration of antibiotic therapy required to achieve cure. This indolent response has been partly attributed to the ability of subpopulations of less metabolically active *Mycobacterium tuberculosis* (*Mtb*) to withstand killing by current anti-TB drugs. We have used immune modulation with a phosphodiesterase-4 (PDE4) inhibitor, CC-3052, that reduces tumor necrosis factor alpha (TNF-α) production by increasing intracellular cAMP in macrophages, to examine the crosstalk between host and pathogen in rabbits with pulmonary TB during treatment with isoniazid (INH). Based on DNA microarray, changes in host gene expression during CC-3052 treatment of *Mtb* infected rabbits support a link between PDE4 inhibition and specific down-regulation of the innate immune response. The overall pattern of host gene expression in the lungs of infected rabbits treated with CC-3052, compared to untreated rabbits, was similar to that described in vitro in resting *Mtb* infected macrophages, suggesting suboptimal macrophage activation. These alterations in host immunity were associated with corresponding down-regulation of a number of *Mtb* genes that have been associated with a metabolic shift towards dormancy. Moreover, treatment with CC-3052 and INH resulted in reduced expression of those genes associated with the bacterial response to INH. Importantly, CC-3052 treatment of infected rabbits was associated with reduced ability of *Mtb* to withstand INH killing, shown by improved bacillary clearance, from the lungs of co-treated animals compared to rabbits treated with INH alone. The results of our study suggest that changes in *Mtb* gene expression, in response to changes in the host immune response, can alter the responsiveness of the bacteria to antimicrobial agents. These findings provide a basis for exploring the potential use of adjunctive immune modulation with PDE4 inhibitors to enhance the efficacy of existing anti-TB treatment.

## Author Summary

Tuberculosis (TB) caused by *Mycobacterium tuberculosis* (*Mtb*) is a leading infectious cause of morbidity and mortality. Although current antibiotic regimens can cure TB, treatment requires at least six months for completion. Recent studies indicate that bacteria in a less metabolically active state are less responsive to antibiotic killing and suggest that this may partly explain the long duration required for TB treatment. In this study, using a rabbit model of pulmonary TB, we show that immune modulation of *Mtb* infected animals with CC-3052, a phosphodiesterase-4 (PDE4) inhibitor that reduces tumor necrosis factor alpha (TNF-α) production by increasing intracellular cAMP levels, resulted in the down-regulation of host genes involved in the innate immune response. Bacteria from the lungs of CC-3052 treated rabbits displayed differential expression of those genes associated with stress responses. In addition, co-treatment of INH with CC-3052 abolished the INH-induced *Mtb* gene expression in the infected rabbits. Importantly, bacillary clearance from the lungs of rabbits co-treated with CC-3052 and INH was improved over that in animals treated with INH alone. The results of this study provide a basis for novel use of immune modulation to improve the efficacy of antibiotic therapy and to shorten the duration of TB treatment.

## Introduction

Despite effective chemotherapy available for over 50 years, and development of a control strategy of directly observed therapy short-course (DOTS), tuberculosis (TB) remains the leading cause of adult mortality attributable to a single infectious disease [1]. *M. tuberculosis* (*Mtb*), the causative agent of TB, is an intracellular pathogen that is well adapted to survive in host phagocytes within lung granulomas in humans and experimentally infected animals [2]–[4]. The outcome of *Mtb* infection is largely determined by a delicate balance between the host immune response and bacterial evasion and/or subversion of this response, resulting in successful control of the infection or manifestations of active disease of different severity [5], [6]. In the presence of an optimal host immune response, growth of the infecting *Mtb* is controlled efficiently and the bacilli often cannot be detected in infected tissues by the conventional colony-forming unit (CFU) assay. However, during growth arrest, not all the infecting bacilli are necessarily killed. Rather, they can adapt to survive in a viable latent state, serving as a reservoir for potential reactivation TB in the host, when immunity weakens [7]–[10]. Following *Mtb* infection of a host with a suboptimal immune response, the bacilli, internalized into the phagosome of macrophages and dendritic cells, replicate and grow [11], [12]. Within the phagosome, *Mtb* must resist the bactericidal molecules of the host cell, including abundant reactive oxygen (ROS) and nitrogen species (RNS), hydrolytic enzymes and an acidic pH [13]–[15]. The invading bacteria counteract this hostile host environment by dampening the process of phagosome maturation, inhibiting lysosome-phagosome fusion and limiting acidification, and shifting from a reliance on high oxygen and a predominantly carbohydrate carbon source for growth. Several mycobacterial genes involved in the bacterial response to phagosomal stresses, such as hypoxia, starvation, iron depletion, acid shock and alternate carbon metabolism, have been reported to contribute to the metabolic adaptation of the bacteria to intracellular survival and growth [16].

Activation of the immune response following *Mtb* infection affects the expression of many host genes that are involved in the production of cytokines, chemokines, surface receptors, and molecules associated with intracellular signaling [6], [17]. These early changes affect subsequent cellular events, including extravasation of leukocytes from the circulation, migration of immune cells to affected tissues and lymphoid organs, and proliferation of effector cells of the innate and adaptive immune response, in an orchestrated response to fight the infection [18]–[20]. An association between the level of macrophage activation and *Mtb* intracellular survival has been reported previously [12], [21]. In particular, RNS produced by activated macrophages inhibit the growth of *Mtb* and have been implicated as an environmental cue directing the physiologic shift of the bacilli towards a state of dormancy [10], [22], [23]. Thus, both the host cells and the bacilli alter their gene expression during *Mtb* infection. However, the specific nature and the interdependence of these events, and the links between the host-pathogen crosstalk and the outcome of *Mtb* infection, are not fully understood.

Tumor necrosis factor-alpha (TNF-α), produced by activated macrophages and other cells of the immune system, is required for the protective host response against *Mtb* infection. This inflammatory cytokine renders the macrophages more capable of controlling the growth of or killing intracellular *Mtb* [24]–[26]. In addition, TNF-α plays a vital role in coordinating and driving the host inflammatory response [27]. While complete inhibition of TNF-α production results in exacerbation or reactivation of TB in animals and humans, excessive TNF-α levels can lead to severe inflammation and damage to host cells and tissues [28], [29]. Since intracellular cyclic AMP (cAMP) regulates TNF-α production, the total cellular TNF-α level and the resulting tissue inflammation are determined in part by the level of this molecule [30]. Consequently, agents that increase cellular cAMP levels have anti-inflammatory properties [31]–[33]. Consistent with these findings, inhibitors of PDE4 (PDE4i) that increase cellular cAMP levels have been shown to reduce TNF-α production and dampen inflammation [34]–[36].

Pharmacologic inhibition of TNF-α production has recently been considered as a therapeutic modality in inflammatory diseases [37]–[39]. Inhibition of TNF-α production by thalidomide treatment in vivo and in vitro has been reported earlier [40], [41]. Additional studies have shown that thalidomide treatment, in combination with antibiotics, reduces TNF-α production in patients with pulmonary TB and can improve treatment outcome [42]. To avoid the side effects of thalidomide, analogs of the drug have been synthesized and screened for their safety and TNF-α inhibitory capacity [43], [44]. One class of synthetic compounds acts as effective PDE4i, which reduce TNF-α production by increasing intracellular cAMP levels. These PDE4i are non-emetic, non-teratogenic, and several of them have been found to be well tolerated by humans in Phase I and II clinical studies [45]. One of these PDE4i, used in the present study, CC-3052, is water soluble, more stable in human plasma and ∼200-fold more potent in reducing TNF-α production, compared to the parent drug [46]. In peripheral blood mononuclear cells (PBMC), the effect of CC-3052 on TNF-α inhibition is dose-dependent [46]. Importantly, at pharmacologically active doses, CC-3052 affects mainly monocyte and macrophage TNF-α production, i.e. the innate immune response, and does not have a significant effect on T cell activation, suggesting a highly cell-specific mechanism of action [47]–[49].

Isoniazid (INH) is one of the first-line antibiotics used in TB treatment. Though INH efficiently kills actively growing *Mtb*, dormant and non-replicating bacilli are killed only poorly [50], [51]. This observation has been reported in humans, mouse and guinea pig models of experimental pulmonary TB, as well as in vitro, using broth culture and *Mtb* infected macrophages [52], [53]. Exposure to INH results in the expression of a number of *Mtb* genes involved in multiple stress and/or toxic responses [54]–[56]. In addition, a specific group of INH-responsive *Mtb* genes, involved in fatty acid metabolism and cell wall biosynthesis, have been reported to be differentially expressed between actively growing versus dormant bacilli, during acute and chronic mouse infection, respectively [57]. These findings highlight the close links between the regulation of *Mtb* gene expression, bacterial metabolism and the response of the bacilli to INH treatment.

As mentioned above, activation of macrophages by *Mtb* infection creates a more hostile intracellular environment for the bacilli to survive and grow [18]–[20]. It has been postulated that robust macrophage activation drives a sub-population of infecting organisms into a metabolic state that is recalcitrant to antibiotic killing [10], [22], [23]. Consequently, we hypothesize that dampening macrophage activation will alleviate the stress on the intracellular bacilli, thereby creating a more permissive environment for bacterial metabolism, resulting in improved efficacy of antibiotic killing. To test this hypothesis, using a rabbit model of pulmonary *Mtb* infection, we examined the impact of CC-3052 treatment on the interactions between the host and pathogen, as revealed by the changes in the expression of host and pathogen genes. We used the rabbit model because, upon *Mtb* infection, rabbits develop progressive pulmonary cavitary TB that, in contrast to the mouse, is similar to the pathologic process seen in humans. We determined which immune response genes in the infected rabbit were specifically affected by treatment with CC-3052. In addition, we evaluated corresponding changes in the expression profile of bacterial genes, with particular emphasis on the stress and INH response genes. Finally, we examined the impact of CC-3052 treatment on bacillary load in the lungs of *Mtb* infected rabbits with and without INH co-treatment. The results of our study provide data to support the idea that combining anti-TB drugs with an adjunctive immune modulator may enhance the efficacy of current TB therapy regimens and shorten the duration of treatment if applied appropriately to humans.

## Results

### Effect of CC-3052 treatment on differential host gene expression in *Mtb* infected rabbit lungs

Owing to the current paucity in immunologic reagents to analyze the cellular function and soluble mediators of immunity in rabbits, we used microarray technology to investigate the rabbit immune response to pulmonary *Mtb* infection. Ingenuity Pathway Analysis (IPA) was used for functional classification and pathway construction of differentially expressed rabbit genes during *Mtb* infection and CC-3052 treatment. Since an annotated rabbit genome database for functional analysis is currently unavailable, we utilized the homologues of rabbit genes from the annotated human, mouse and rat genomes available in the IPA knowledge base for our analysis. Rabbits were infected with *Mtb* by the respiratory route, and the development of the host immune response in the lungs was evaluated at 4, 8 and 12 weeks post-infection using microarrays (Table 1). Of the 43,603 oligonucleotide probes represented on the rabbit array and analyzed by global gene expression patterns, 5,332 were significantly differentially expressed (3,075 up; 2,257 down) in response to 4 weeks of *Mtb* infection of the lungs, compared to uninfected, naïve animals (significant change defined as ±2 fold with *p*≤0.05). Progressive up-regulation of host genes was noted as the infection progressed for 8 weeks and 12 weeks, with 8,597 (5,344 up; 3,253 down) and 13,783 (4,260 up; 9,523 down) genes differentially regulated, respectively. It is important to note that with time, as the granulomas mature and differentiate following *Mtb* infection in rabbit lungs, the profile of gene expression also changes. In addition, of the total number of differentially regulated genes only a subset overlap (1,269, 5,212 and 1,584 genes respectively for 4 vs. 8, 8 vs. 12 and 4 vs.12 weeks, data not shown), consistent with the histologic observation of heterogeneity of the differentiating granulomas [58].

**Table 1:** Number of differentially expressed host genes in *Mtb* infected rabbit lungs with or without CC-3052 treatment.

		Number of differentially expressed genes*		
*Time p.i. (wk)*	*CC-3052 treatment (wk)*	*Total*	*Up*	*Down*
4	0	5332	3075	2257
8	0	8597	5344	3253
8	4	1055	34	1021
12	0	13783	4260	9523
12	8	1272	117	1155

*Selected with a cut-off of +/−2 fold and p≤0.05 among 43,603 probes in the microarray slide.

CC-3052 treatment was started at 4 weeks post-infection, and lung tissue was collected after 4 and 8 weeks of treatment (i.e. at 8 and 12 weeks post-infection). To study the changes in host gene expression induced by CC-3052 treatment, we compared the gene expression profiles in the *Mtb* infected lungs of CC-3052 treated rabbits to untreated infected control animals at the same time points. The complete list of rabbit genes differentially expressed by CC-3052 treatment at the time points tested has been submitted to the Gene Expression Omnibus (GEO) database (GSE27992). Among the rabbit genes differentially expressed by *Mtb* infection, statistically significant changes in the expression of 1,055 (34 genes up; 1,021 down) and 1,272 (117 genes up; 1,155 genes down) genes were observed after 4 and 8 weeks of CC-3052 treatment, respectively (Table 1). Expression of 20 overlapping genes was affected at both 4 (1 up; 19 down) and 8 weeks (9 up; 11 down) of CC-3052 treatment. The distribution, among various cellular immune response pathways, of the genes differentially expressed in response to 4 and 8 weeks of CC-3052 treatment, is shown in Table 2. About 30% of the genes differentially expressed following 4 weeks of CC-3052 treatment, compared to no treatment, were involved in host immune response-related pathways. These included immune cell growth and proliferation, cell morphology and movement, cell death/apoptosis, immune cell trafficking and hematologic development and function (Table 2). At 8 weeks of CC-3052 treatment, somewhat higher numbers of genes, involved in immune cell growth/proliferation, cell death, molecular transport, hematologic development/function, cell trafficking, were modulated compared to 4 weeks of treatment. In contrast, the number of rabbit genes involved in tissue morphology/development and immune cell signaling/interaction were reduced at 8 weeks compared to 4 weeks of CC-3052 treatment. In summary, the microarray analysis reveals differential expression of many host genes during *Mtb* infection in the rabbit lung as well as the modulation of a subset of those genes, involved in key host immune response pathways, following CC-3052 treatment.

**Table 2:** Classification of differentially expressed host genes in *Mtb* infected rabbit lungs treated with CC-3052.

	Number of genes affected					
	4w CC-3052 (8wpi)			8w CC-3052 (12wpi)		
*Function/Pathway*	*Total*	*Up*	*Down*	*Total*	*Up*	*Down*
Cell Growth/Proliferation	64	15	49	72	11	61
Cell Death	61	12	49	64	10	54
Molecular Transport	34	13	21	54	6	48
Tissue Morphology/Development	75	12	63	37	8	29
Hematologic Development/Function	43	17	26	46	6	40
Immune Cell Trafficking	31	10	21	33	6	27
Respiratory Disease	29	9	20	33	9	24
Cell Signaling/Interaction	43	18	25	38	8	30

### Modulation of the TNF-α network genes by CC-3052 treatment in *Mtb* infected rabbit lungs

**Figure 1:**
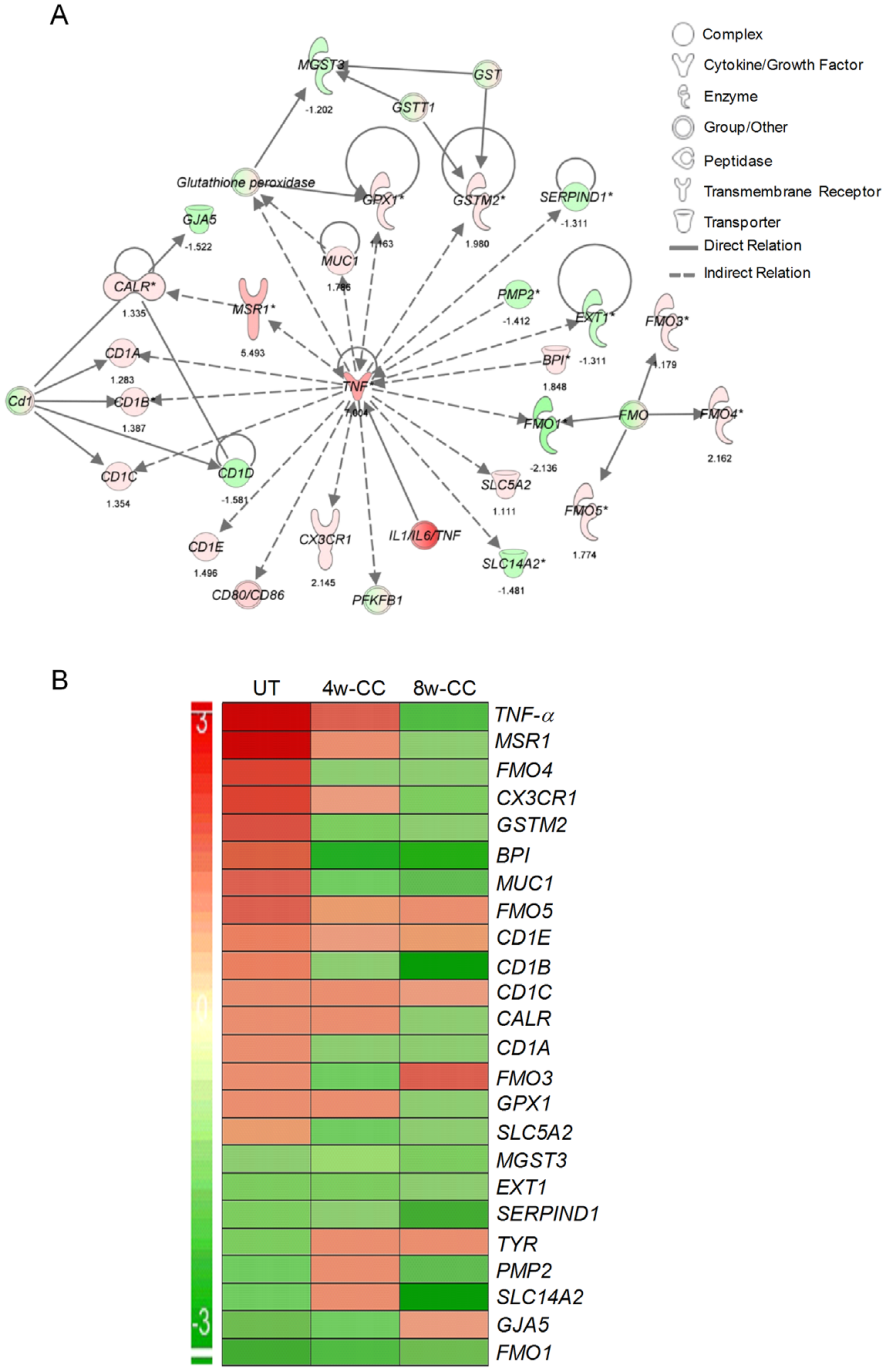
Differential expression of genes involved in the TNF-α network between untreated and CC-3052 treated, *Mtb* infected rabbits. The absolute expression level of rabbit genes, from the microarray experiments on the lungs of *Mtb* infected and CC-3052 treated or untreated rabbits, was used in Ingenuity Pathway Analysis (IPA) software program to construct pathway maps. (A) Pathway map of rabbit genes regulating- or regulated by- TNF-α at 4 weeks (No CC-3052 treatment) The numbers at the bottom of each gene represent the absolute expression levels. (B) Intensity plot of TNF-a network genes before (UT) or after 4weeks (4w-CC) or 8 weeks (8w-CC) of CC-3052 treatment. For both A and B, green color represents down-regulation and red color represents up-regulation. The level of up- or down-regulation is related to the intensity of respective colors.

Since inhibition of PDE4 reduces *TNF-α* expression, a key regulator of innate and acquired immunity, we studied the effect of CC-3052 treatment on the genes of the TNF-α network. The expression patterns of a subset of genes that directly regulate or are regulated by TNF-α, in *Mtb* infected rabbit lungs, at 0, 4 and 8 weeks of CC-3052 treatment were analyzed by microarray and compared to levels of expression in lungs of infected untreated animals (Figure 1). The fold changes in gene expression between CC-3052 treated and untreated animals were used for the pathway construction using IPA. In order to determine absolute expression levels, we set no cut-off values for the expression levels of individual genes in the network.

 In the untreated rabbits, *Mtb* infection up-regulated more than half of the genes (18 out of 32) in the TNF-α network, including *TNF-α* itself (Figure 1A). Only 14 out of 32 genes were down-regulated at 4 weeks post-infection compared to uninfected, control rabbits. After 4 and 8 weeks of CC-3052 treatment, the level of expression of the majority of these genes, specifically those involved in tissue inflammation and the innate immune response, including *TNF-α*, were progressively reduced (about 54% of genes at 4 weeks and 75% at 8 weeks of CC-3052 treatment) compared to untreated animals (Figure 1B). Not all of the genes in the TNF-α network were affected by CC-3052 treatment. Eight genes that are indirectly related to the TNF-α network (*CD1c*, *CD1e*, *FMO-1*, *EXT-1*, *MGST-3*, and *SERPIN-D1*) were expressed at comparable levels in untreated and CC-3052 treated, infected rabbits at 4 and 8 weeks post-treatment. Overall, the expression pattern of key genes of the TNF-α network impacted by CC-3052 treatment suggests a gradual reduction in the activation of the TNF-α network in the lungs of *Mtb* infected rabbits.

### Effect of PDE4 blockade on expression of representative host genes in *Mtb* infected rabbit lungs

**Figure 2:**
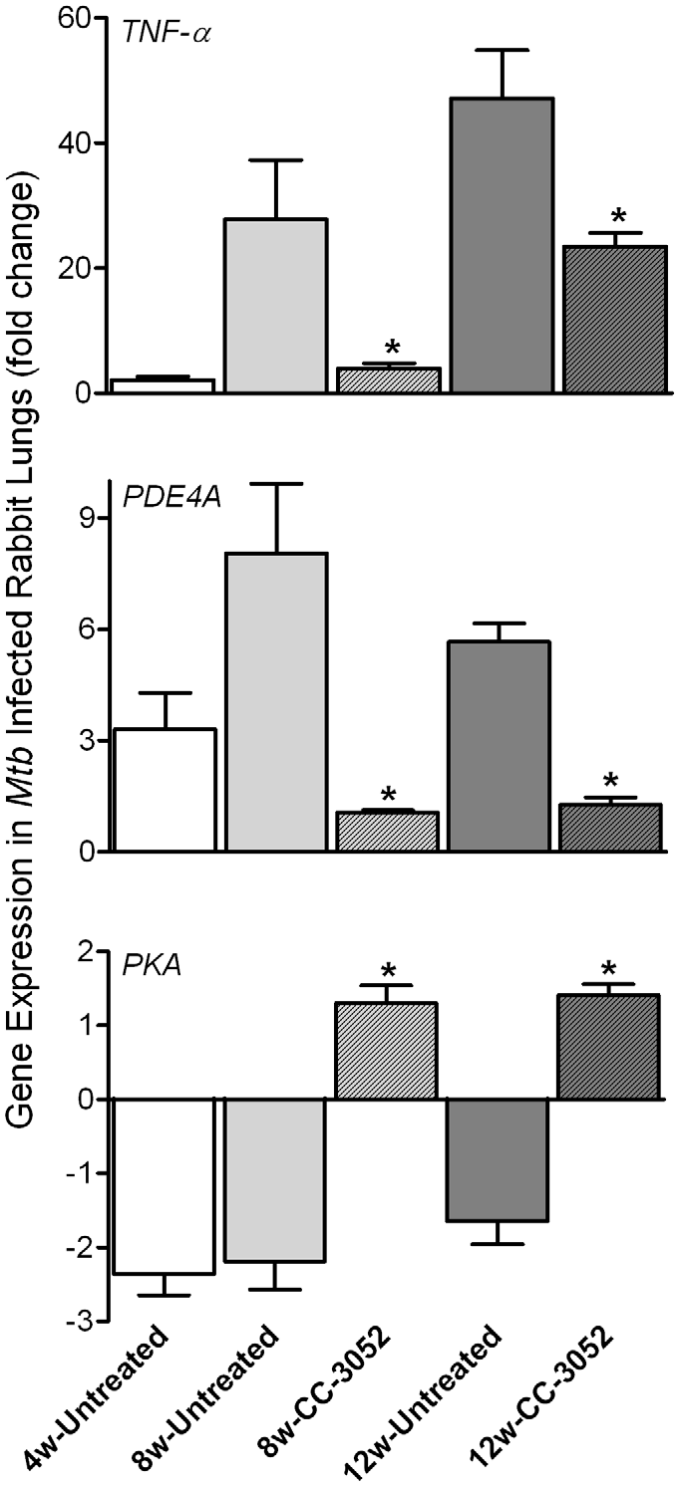
qRT-PCR of rabbit genes that are directly related to the mode of action of CC-3052. Total host and bacterial RNA was isolated from lungs of the rabbits from various treatment groups (mentioned in the Materials and Methods section) of *Mtb* infected and uninfected rabbits at 4, 8 and 12 weeks post-infection and used to enumerate the transcript levels in qRT-PCR experiments with gene specific primers. The difference in specific gene expression was calculated as fold change in expression between CC-3052 treated vs. untreated or *Mtb* infected vs. uninfected rabbits at each time point. The X-axis legend is common for all four figures. Changes in the rabbit gene expression by *Mtb* infection are represented as relative fold change, compared to the levels in uninfected rabbit lungs, and normalized to housekeeping *GAPDH* levels. Results shown are mean ± standard deviation from at least 2 experiments done in duplicate (total of at least 4 data points) with samples from two to four animals per group per time point. *Statistically significant difference between CC-3052 treated and untreated animals at the same time point (*p*≤0.05).

To evaluate the impact of CC-3052 treatment on the expression of host genes associated with PDE4, the target of CC-3052, we analyzed mRNA levels from the lungs of *Mtb* infected rabbits by quantitative real-time PCR (qRT-PCR) (Figure 2). At 4 weeks post-infection, low basal levels of expression of *TNF-α* and *PDE4A* were noted in comparison with uninfected animals. *TNF-α* mRNA levels increased to about 30- and 45-fold, respectively, in response to 8 and 12 weeks of infection, but were not significantly different between these two time points. The expression of *PDE4A* was similarly elevated at 8 weeks and was somewhat lower at 12 weeks post-infection. Treatment of infected rabbits with CC-3052 (for 4 or 8 weeks) significantly reduced the expression levels of both *TNF-α* and *PDE4A* (Figure 2). On the other hand, while the lungs of *Mtb* infected animals showed lower *PKA* expression at all time points in comparison with uninfected controls, CC-3052 treatment reversed this effect significantly, leading to about 3-fold higher levels of *PKA* mRNA at 4 and 8 weeks of treatment (i.e. 8 and 12 weeks post-infection) (Figure 2).

**Figure 3:**
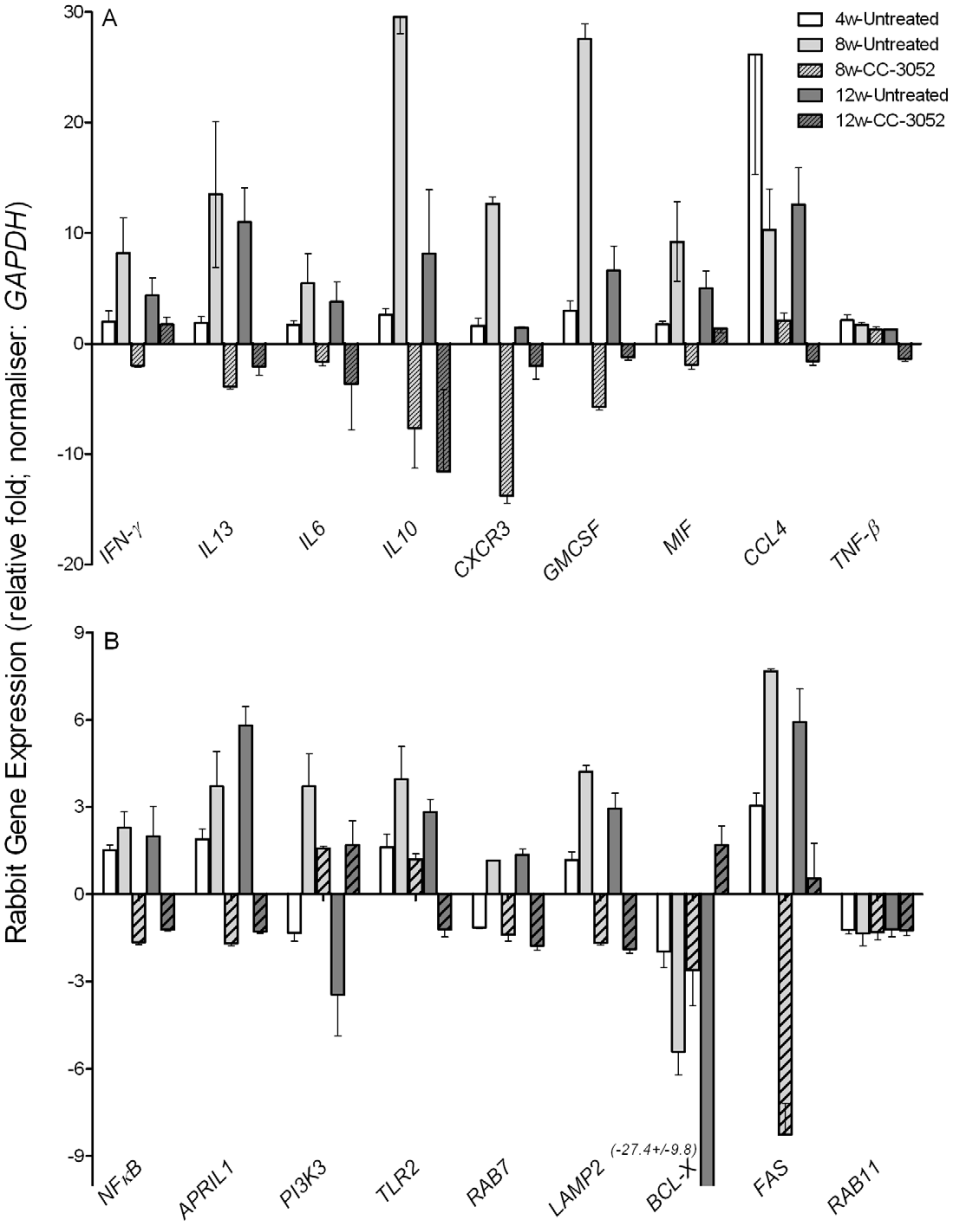
Effect of CC-3052 treatment on rabbit gene expression during *Mtb* infection. Total RNA isolated from the lungs of *Mtb* infected and uninfected rabbits that were either treated with CC-3052 or left untreated were used to determine the abundance of transcripts using qRT-PCR with gene specific primers. (A) Level of expression of rabbit genes encoding for cytokines (*IFN-γ, IL13*, *IL6*, *IL10* and *TNF-β)* and chemokines (*CXCR3*, *GMCSF*, *MIF* and *CCL4*). (B) Expression level of rabbit genes encoding for intracellular signaling molecules (*NFκB*, *APRIL1*, *PI3K3* and *TLR2*), endosomal trafficking markers (*RAB7*, *LAMP2* and *RAB11*) or apoptosis pathways (*BCL-X* and *FAS*) in the lungs of *Mtb* infected rabbits. Change in gene expression levels in the CC-3052 treated rabbits were calculated as relative fold difference after normalization to the level of expression of the housekeeping gene, *GAPDH*, and calibrated against the levels in uninfected or untreated animals. The difference in the level of expression of all tested genes between CC-3052 treated (CC) and untreated (UT) animals were statistically significant (*p*≤0.05) at 8 and 12 weeks post-infection except for the following: *TNF-β*at 8 weeks and *IFN-γ* at 12 weeks. Expression of *RAB11* was not statistically different at both 8 and 12 weeks post-infection. The results shown are mean ± standard deviation from at least 2 experiments done in duplicate (total of at least 4 data points) with samples from two to four animals per group per time point.

We then evaluated by qRT-PCR the mRNA levels of a subset of genes, including cytokines (*IFN-γ*, *IL10*, *IL13*, *IL6* and *TNF-β*), growth factors, chemokines and their receptors (*CXCR3*, *GMCSF*, *MIF* and *CCL4*), signaling molecules and receptors (*NFκB*, *APRIL1*, *PI3K3* and *TLR2*) and intracellular trafficking and apoptosis-related genes (*RAB7*, *LAMP2*, *BCL-X*, *FAS* and *RAB11*) (Figure 3A and B). Many of these gene products are known markers of macrophage activation and maturation, endosomal trafficking and/or Th1/Th2 immunity [59]–[60]. In untreated rabbits, most of these genes were progressively induced by *Mtb* infection from 4 to 8 weeks, and many had reached a plateau (*IFN-γ, IL13, IL6, MIF, CCL4, NFκB, APRIL, TLR2, RAB7 and LAMP2*) or were even reduced (*IL10*, *CXCR3*, *GMCSF* and *PI3K3*) by 12 weeks post-infection. In contrast, expression of the anti-apoptotic gene *BCL-X* was progressively down-regulated by infection, and there were no significant changes in the expression of other selected genes, including *TNF-β* and *RAB11* (Figure 3A and B). Following 4 or 8 weeks of CC-3052 treatment, the expression of many of these same genes were significantly reduced in comparison to *Mtb* infected untreated animals. The expression of genes that were not regulated by *Mtb* infection, for example *RAB11* and *TNF-β*, were also unaffected by CC-3052 treatment (Figure 3). In summary, the expression profile of the host immune response genes in the lungs of *Mtb* infected rabbits suggests that CC-3052 treatment of rabbits with pulmonary TB resulted in a dampening of macrophage activation in the lungs.

### Effect of CC-3052 treatment on *Mtb* gene expression in infected rabbit lungs

**Figure 4:**
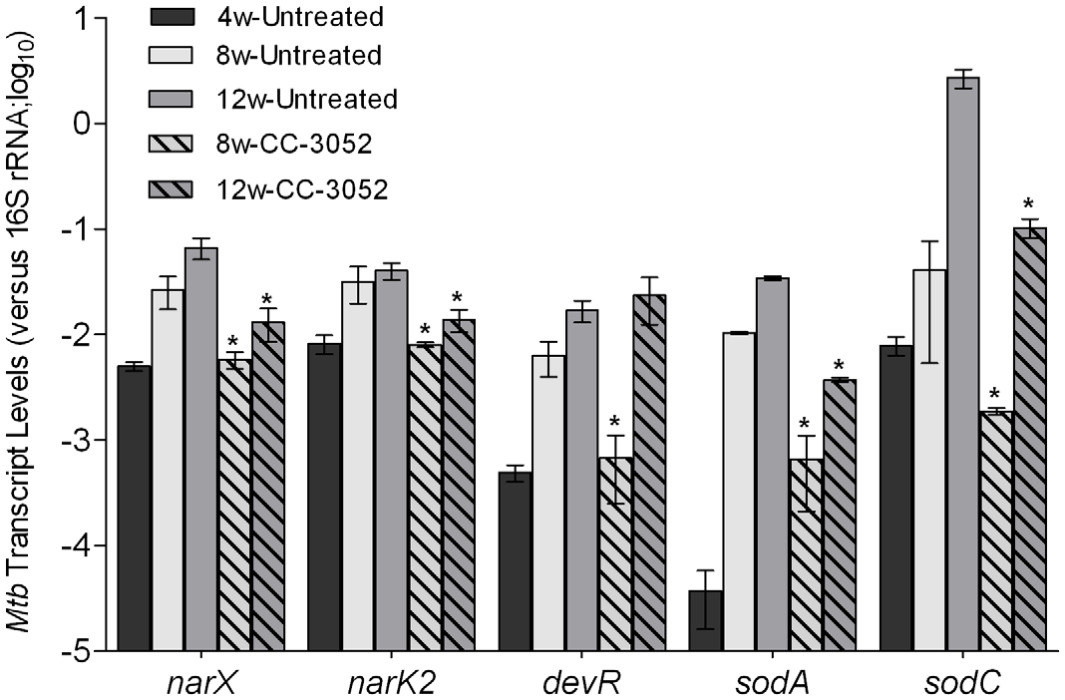
Effect of CC-3052 treatment on the expression of *Mtb* stress response genes in rabbit lungs. Total *Mtb* RNA was isolated from the lungs of rabbits from various treatment groups (mentioned in the Materials and Methods section) of *Mtb* infected rabbits at 4, 8 and 12 weeks post-infection and used to quantify the amount of transcripts in qRT-PCR experiments with primers specific for those *Mtb* genes that are involved in the stress response. The values plotted in the graph are absolute transcript levels of each *Mtb* gene after normalization to 16S rRNA levels. The mycobacterial genes represented in the graph are: *narX*- probable nitrate reductase; *narK2*- possible nitrate/nitrite transporter; *devR*- two component transcriptional regulatory protein; *sodA*- superoxide dismutase A; *sodC*-superoxide dismutase C. The results are shown as mean ± standard deviation from at least 2 experiments done in duplicate (total of at least 4 data points) with samples from two to four animals per group per time point. *Statistically significant difference between CC-3052 treated and untreated animals (*p*≤0.05).

We analyzed the impact of CC-3052 treatment on gene expression of the infecting bacilli. Total RNA from *Mtb* isolated from infected rabbit lungs was prepared, and the expression levels of selected hypoxic/oxidative stress response genes (*narX*, *narK2*, *devR*, *sodA* and *sodC*) were evaluated by qRT-PCR. Expression of these genes was induced progressively in the rabbit lungs from 4 to 12 weeks post-infection, with the exception of *narK2* which reached a plateau after 8 weeks (Figure 4), consistent with our observation of progressive activation of macrophages in the granulomas. This notion is also supported by previous demonstrations that rabbit granulomas are hypoxic [61] and exposure of *Mtb* to hypoxia differentially regulates several genes of the *dosR* regulon [62], [63]. Treatment of *Mtb* infected rabbits with CC-3052 significantly reduced the expression of *narX*, *narK2*, *devR*, *sodA* and *sodC* from 5- to 150- folds (*p*<0.05), compared to infected but untreated animals. These results are consistent with our hypothesis that CC-3052-mediated reduction in macrophage activation would be accompanied by changes in the extent of the *Mtb* stress response during intracellular survival (Figure 4). However, treatment of log-phase *Mtb* culture with equimolar amount (compared to in vivo) of CC-3052 had no impact on the mRNA levels of these and other dormancy/stress response-related *Mtb* genes, compared to untreated controls (Supporting Information Figure S1), indicating that the effect of CC-3052 on *Mtb* gene expression was specific to the in vivo conditions.

**Figure 5:**
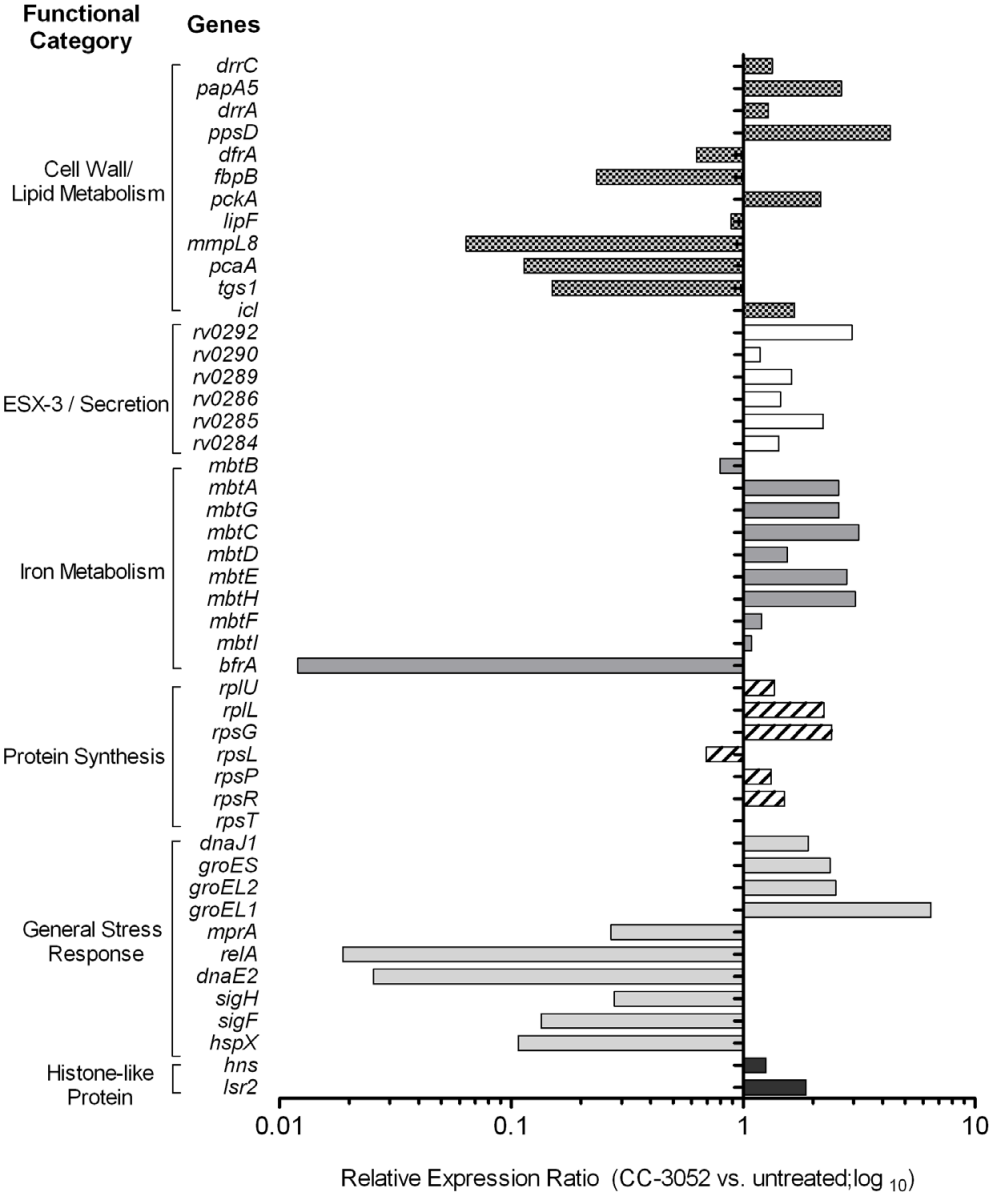
Effect of CC-3052 treatment on the expression of *Mtb* metabolic genes in rabbit lungs. Total *Mtb* RNA was isolated from the lungs of rabbits from various treatment groups (mentioned in the Materials and Methods section) of *Mtb* infected rabbits at 8 weeks post-infection and used to measure the transcript levels using qRT-PCR with primers specific for *Mtb* genes involved in various metabolic processes. The change in *Mtb* gene expression is represented as relative ratio between untreated and CC-3052 treated samples, after the levels of expression of each gene was normalized to 16S rRNA levels. Description of individual *Mtb* genes is included in Table S1. Results shown are average values from at least 2 experiments done in duplicate (total of at least 4 data points) with samples from two to four animals per group per time point.

Recently, a comprehensive analysis of the gene expression profiles of *Mtb* isolates, representing diverse lineages, grown in vitro within activated and resting murine macrophages was reported [64]. We selected for study a subset of genes from the Homolka et.al. report, including genes previously reported as differentially expressed by *Mtb* in response to environmental stresses, such as metabolic adaptation to dormancy [65], [66]. The genes were functionally grouped as follows: a) protein synthesis (*rpsT, rpsR, rpsP, rpsL, rpsG, rplL* and *rplU*); b) iron metabolism (*mbtI, mbtF, mbtH, mbtE, mbtD, mbtC, mbtG, mbtA, mbtB* and *bfrA*); c) cell wall/lipid metabolism (*ppsD, drrA, papA5, dfrA, fbpB, pckA, lipF, mmpL8, pcaA, tgs1, icl* and *drrC*); d) general stress response (*hspX, sigF, sigH, dnaE2, relA, mprA, groEL1, groEL2, groES* and *dnaJ*); e) ESX-3/secretion system (*Rv0284-Rv0286, Rv0289, Rv0290* and *Rv0292*); and f) histone-like proteins (*hns* and *lsr2*) (Figure 5). Comparative gene expression profiling revealed that many of the *Mtb* genes involved in protein synthesis, ESX-3/secretion, iron metabolism and histone-like proteins were up-regulated by 4 weeks of CC-3052 treatment in rabbit lungs (Figure 5). In contrast, many of the general stress response and cell wall/lipid metabolism associated genes were down-regulated by CC-3052 treatment. When the *Mtb* gene expression pattern from the lungs of CC-3052 treated rabbits was compared to the functional classifications of Homolka et.al., the bacteria from the rabbits showed a gene expression profile closer to that seen in resting, rather than activated, macrophages (Figure 5) [13], [64], [67]. Thus, our functional analysis of the *Mtb* gene expression profile in the lungs of infected rabbits during CC-3052 treatment suggested that the bacilli were in a different metabolic state than in the lungs of untreated, control rabbits, and that this metabolic state corresponded with that observed in suboptimally activated macrophages.

### Effect of CC-3052 treatment on INH-mediated *Mtb* killing in infected rabbit lungs

**Figure 6:**
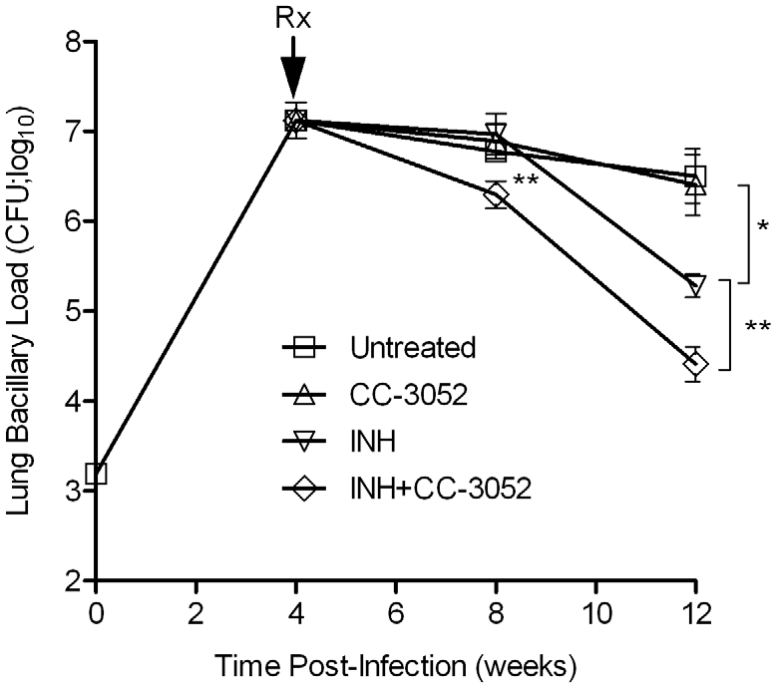
Effect of CC-3052 and INH treatment on the growth of *Mtb* in rabbit lungs. Rabbits were infected with *Mtb* HN878 by aerosol challenge to implant 3.2 log_10_ bacilli on day 0. Starting at 4 weeks post-infection, groups of rabbits were treated with CC-3052, INH, both or left untreated, and treatment continued until 12 weeks post-infection. At 0, 4, 8 and 12 weeks post-infection, groups of 2–4 rabbits per treatment condition, were necropsied and segments of lung tissue were homogenized and plated on 7H9 agar plates to enumerate the numbers of CFU (detailed in the Materials and Methods section). The total numbers of CFU (in log_10_ scale) was calculated for the entire rabbit lungs. Rx represents the starting time point (4 weeks) for CC-3052 and/or INH treatment. The experiment was repeated at least twice with 2–4 animals per time point per treatment group. Results shown are means ± standard deviation from 6–11 animals. *Statistically significant difference between untreated and INH treated animals at 12 weeks post-infection (*p*<0.05). **Statistically significant difference between INH plusCC-3052- and INH alone- treated animals at 8 and 12 weeks post-infection (*p*<0.05).

To study the effect of CC-3052 treatment on the ability of INH to kill *Mtb* in the lungs of infected rabbits, animals were treated from 4 weeks post-infection with high dose INH (50 mg/kg body weight/day) or CC-3052 (25 mg/kg body weight/day), neither or both for 4 or 8 weeks (i.e., 8 or 12 weeks post-infection). In the untreated animals, implantation of about 3.2 log_10_ bacilli (on day 0) into the lungs resulted in progressive, active disease with exponential bacterial growth up to 4 weeks of infection, after which the bacterial counts stabilized and then declined slightly. The control of bacillary load in the lungs of rabbits was not significantly affected by CC-3052 treatment, as indicated by the similar numbers of CFU in untreated and CC-3052 treated animals at all time points tested (Figure 6). There were also no significant differences in numbers of CFU in the liver and spleen between these two groups (data not shown). INH treatment of infected rabbits did not significantly reduce the CFU numbers in the lungs of treated rabbits for the first 4 weeks. However, 8 weeks of INH treatment (i.e. 12 weeks post-infection), the bacillary load in the lungs was reduced by about 1 log_10_ (Figure 6).

**Figure 7:**
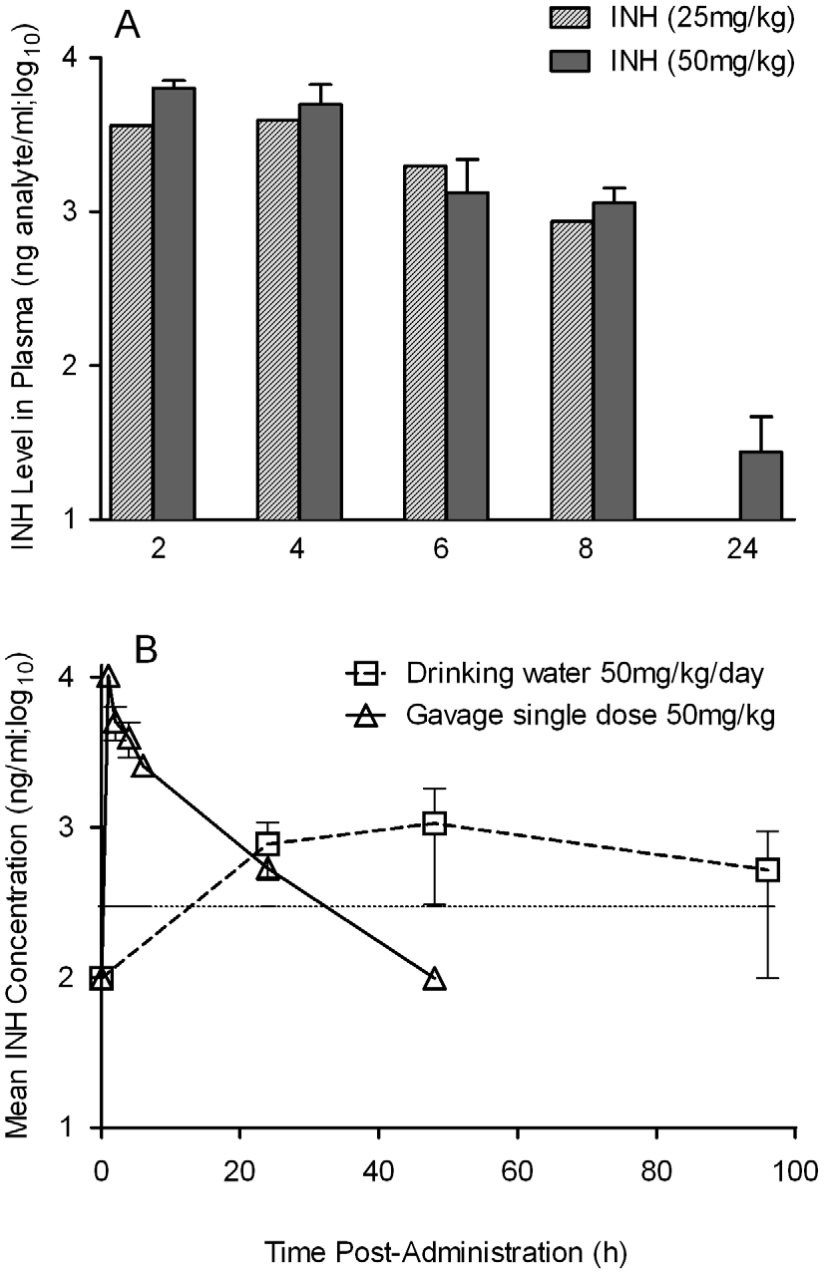
Pharmacokinetics and dynamics of INH uptake into the serum of treated rabbits. (A) Level of INH detected in the plasma of rabbits treated with a bolus dose of either 25 or 50 mg/kg of INH by gavage. INH levels were estimated by HPLC-mass spectrometry as explained in the Materials and Methods section (B) Effect of different modes of administration of INH on rabbit plasma INH levels. Rabbits were treated with INH either by gavage using a rubber feeding tube, or in drinking water. Plasma INH from treated rabbits was separated from the blood collected at indicated time points, post-administration, to measure the INH concentration by HPLC-mass spectrometry as explained in the Methods section. The horizontal dotted line in (B) represents the optimal level of quantification of INH.

The limited efficacy of INH monotherapy in *Mtb* infected rabbits during the first 4 weeks prompted us to evaluate the bioavailability of the drug. To assess the extent to which INH was absorbed into the circulation, we measured the plasma INH concentration in animals that received either 25 mg/kg or 50 mg/kg of INH per day by gavage administration. Both doses of the drug resulted in similar plasma levels up to 8 hours post-administration (Figure 7A). However, only the 50 mg/kg dose of INH resulted in detectable INH levels in the plasma up to 24 hours. To compare INH availability when administered in drinking water to that of the gavage route of delivery, rabbits were treated with 50 mg/kg of INH by either route, and the pharmacokinetics of INH in the plasma were compared (Figure 7B). The parameters for INH in drinking water were calculated, assuming a steady state dosing during a 24-hour interval. Compared to INH in drinking water, a single dose of 50 mg/kg of INH administered by gavage showed about 100-fold increase in plasma drug levels within 2 hours of administration. However, both routes of administration showed similar INH levels in the plasma at 24 hours post-administration and similar bioavailability, as measured by the area under the curve (Figure 7B). This observation supported the use of gavage administration for appropriate dosing of INH, thereby avoiding the uncertainty of drug dosing through drinking water which arises from the fact that rabbits do not consume reproducible volumes of water daily.

When rabbits were treated with CC-3052 in combination with INH for 4 weeks, a statistically significant reduction in the numbers of CFU was noted, compared with INH alone (*p* = 0.027, Figure 6). An additional 4 weeks of co-treatment resulted in a 10-fold reduction in bacillary load compared to INH alone (*p* = 0.003, Figure 6). Thus, while the rate of *Mtb* killing was similar during the second month of treatment, the absolute numbers of CFU in CC-3052 co-treated animals were significantly lower than in the INH alone group by the end of experiment. It is important to note that treatment of infected rabbits with INH alone or in combination with CC-3052 for up to 2 months did not produce INH resistant bacilli, as determined from the CFU assay by plating the lung homogenates in the presence and absence of INH. The effect of CC-3052, either alone or in combination with INH, on *Mtb* was specific to the in vivo conditions present in the lungs of infected rabbits, since the addition of up to 50X molar excess of CC-3052 and 0.2 µg/ml of INH (the minimal inhibitory concentration) to a growing *Mtb* culture in vitro did not affect the growth or INH killing significantly (Figure S2). Future experiments, including prolonging the duration of treatment of infected rabbits (more than 8 weeks) and varying the starting time of treatment, will determine if combined administration of INH and CC-3052 can fully eliminate the bacteria from rabbit lungs.

### Effect of CC-3052 treatment on INH-mediated *Mtb* gene expression in infected rabbit lungs

**Figure 8:**
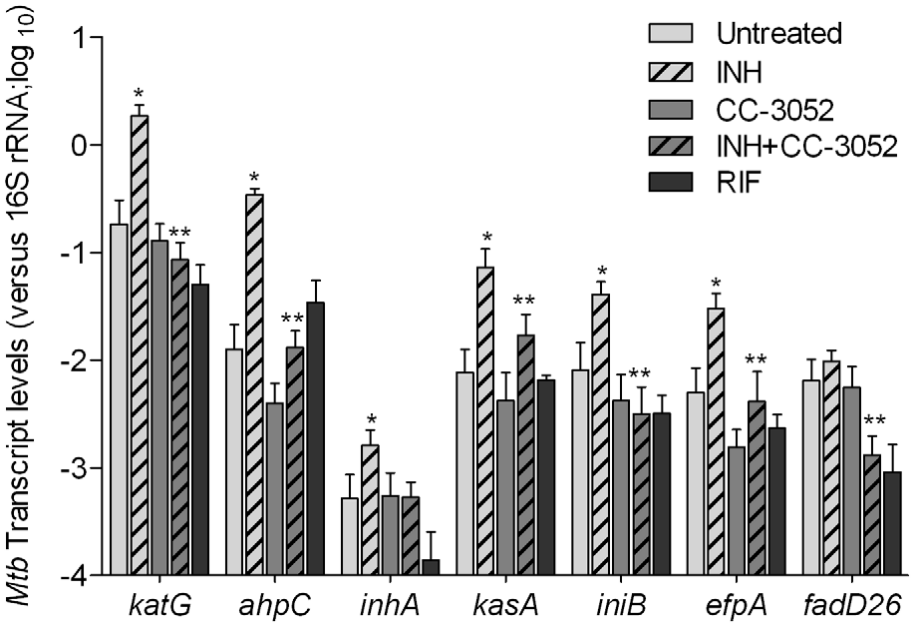
Effect of CC-3052 treatment on the expression of *Mtb* INH responsive genes in rabbit lungs. Total *Mtb* RNA was isolated from the lungs of rabbits from various treatment groups (mentioned in the Materials and Methods section) of *Mtb* infected rabbits at 8 weeks post-infection and used to quantify the amount of transcripts by qRT-PCR with primers specific for those *Mtb* genes that respond to exposure to INH. The difference in *Mtb* gene expression was calculated as relative ratio (in log_10_ scale) between untreated and CC-3052 treated rabbits, after the level of expression of each gene was normalized to 16S rRNA levels. RIF represents samples from rabbits treated with rifampicin. The mycobacterial genes represented in the graph are: *katG*- catalase-peroxidase-peroxynitritase; *ahpC*- alkyl hydroperoxidase C; *inhA*- NADH-dependant enoyl-ACP reductase; *kasA*- beta-ketoacyl-ACP-synthase; *iniB*- isoniazid inducible gene; *efpA*- possible integral membrane efflux protein; *fadD26*- fatty acid CoA synthase. Results shown are mean ± standard deviation from at least 2 experiments done in duplicate (total of at least 4 data points) with samples from two to four animals per group per time point. * Statistically significant difference between untreated and INH treated animals (*p*<0.05). ** Statistically significant difference between INH plusCC-3052- and INH alone- treated animals (*p*<0.05).

We next analyzed the impact of INH treatment on the expression of INH-associated *Mtb* genes in the presence and absence of CC-3052 in rabbit lungs (Figure 8). Total *Mtb* RNA was isolated from the lungs of infected rabbits and analyzed by qRT-PCR. Similar to results reported in several studies, after 4 weeks of INH treatment, we observed significant increases in the mRNA levels of the *Mtb* genes, *katG, ahpC, inhA, kasA, iniB* and *efpA* (Figure 8). [55], [57], [68]. There was no statistically significant differences in the level of expression of *fadD26* between *Mtb* RNA pools from INH treated and untreated animals. The gene induction profile was specific for INH treatment, since these genes were not affected by treatment of similarly infected rabbits with rifampicin (RIF), another important antibiotic for TB treatment (Figure 8). The fold change in expression for these genes, after normalization to the 16S rRNA, ranged from 2- (for *inhA*) to about 20-fold (for *katG* and *ahpC*) (*p*≤0.05) in comparison to levels observed in untreated animals. The levels of expression of these genes in the lungs of untreated and CC-3052 alone-treated animals were comparable and significantly less than what was observed in the INH only treated animals. Thus, although 4 weeks of INH treatment of infected rabbits did not reduce the bacillary load significantly, exposure to the antibiotic clearly affected the physiology of the infecting bacteria, as manifested by differential gene expression, consistent with previous reports [7], [8], [55], [57], [68]. Interestingly, co-treatment with INH plus CC-3052 reduced the expression of INH–induced genes in *Mtb* to levels similar to those observed in untreated rabbit lungs.

## Discussion

**Figure 9:**
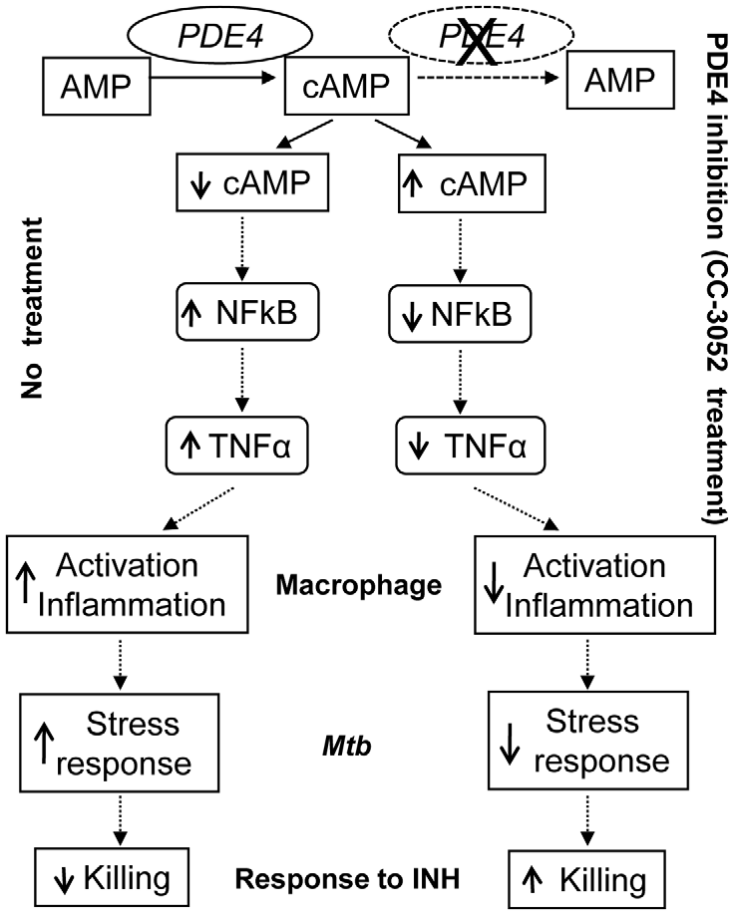
Schematic representation of the mechanism by which CC-3052 treatment affects macrophage activation and improve INH-mediated *Mtb* killing in the lungs of infected rabbits.

In the present study, we describe the effect of PDE4 inhibition on the immune response during *Mtb* infection in a rabbit model of pulmonary TB and show how changes in the expression of immune response genes affect the crosstalk between the host and pathogen (Figure 9). During the course of *Mtb* infection in the lungs of untreated rabbits, expression levels of many host genes involved in the innate immune response were up- regulated. Corresponding changes were observed in the expression of many *Mtb* genes known to be responsible for successful intracellular survival of the bacilli, including genes involved in protecting the bacteria against ROS- and RNS-induced damage and bacillary response to stress induced by nutritional deprivation and acid shock. These genes were up-regulated gradually with disease progression in the lungs of untreated, *Mtb* infected rabbits. Treatment of the *Mtb* infected rabbits with CC-3052 led to significantly reduced expression of many of the host innate immune response genes, including TNF-α and IL-6. Concomitant with these PDE4-induced changes in host gene expression, the levels of expression of many bacterial stress response genes were reduced.

Importantly, CC-3052 treatment of infected rabbits was associated with a loss in the ability of the bacilli to withstand INH killing, and bacillary clearance was improved starting from the first month of treatment compared to INH alone. These results contrast with our previously reported study in the murine TB infection model [49], in which we showed that co-treatment with CC-3052 extended the duration of INH-mediated bacillary clearance from the lungs at later time points, when INH effectiveness began to fail, but did not significantly affect the CFU numbers during early treatment [49]. The differences in the kinetics of improved INH-mediated *Mtb* clearance from the lungs in the presence of immune modulation likely reflect differences in the pathogenesis of *Mtb* infection in these two animal models. While rabbit lungs develop fully matured granulomas by 4 weeks post-infection that progress to necrotizing granulomas and cavities, similar to humans, the mouse immune response to *Mtb* infection involves the accumulation of mononuclear leukocytes that do not differentiate into structured granulomas [69]. Our observations suggest that, in the rabbit, INH fails to kill the bacilli efficiently in the early well-differentiated granulomas of the lungs (4 to 8 weeks post-infection). Moreover, CC-3052 co-treatment improves INH-mediated bacillary clearance at these early time points. Thus, in the present study, we have demonstrated the effect of PDE4 inhibition on *Mtb* killing within a mature granuloma, in contrast to our findings in the mouse, where the impact of immune modulation was observed on residual persisting bacilli towards the end of treatment. It is possible that, in the rabbit model, CC-3052 treatment would further improve *Mtb* killing at later time points (after 12 weeks) when INH alone fails to kill persisters. Indeed, it has been reported that the proportion of physiologically quiescent bacilli and their vulnerability to INH killing determines the overall response to INH treatment [70], [71]. Hence, the differential outcome of INH treatment with and without CC-3052 could be attributed to changes in the metabolism of a sub-population of bacilli [56], [72]. This suggests the presence of a higher percent of bacilli that are dormant and/or non-responsive to INH at 4 weeks post-infection of the rabbit lung, compared to the mouse lung. Since the nature and extent of the host immune response to *Mtb* infection significantly affect the physiology of the infecting bacilli [73], and rabbit granulomas are hypoxic and more mature compared to lesions in the mouse lung, it is expected that the number of bacilli that are non-responsive to INH treatment in the 4-week rabbit granulomas may be relatively high. Taken together, studies in the rabbit provided a useful model that is more similar to human disease than the infection seen in the mouse lung.

The changes observed in host gene expression during CC-3052 treatment of *Mtb* infected rabbits support a link between PDE4 inhibition and specific modulation of the innate immune response. So far, at least 11 families of class 1 PDEs (PDE1-11) have been identified in humans [74]. However, only 3 of the 11 (PDEs 4, 7 and 8) are involved in hydrolyzing cAMP [75]. Of these, PDE4 is the predominant isoform of phosphodiesterase found in monocyte/macrophages [76]. Thus, inhibition of PDE4 would be expected to target monocytes and macrophages, but not T cells and other cells of the immune response. The ability of the rabbits to control bacillary growth in the lungs in the presence of CC-3052 supports the conclusion that this drug does not suppress the acquired immune response. Moreover, in our previous study, the mouse TB model enabled us to directly demonstrate that CC-3052 does not inhibit T cell activation in *Mtb* infected animals [49]. PDE4 hydrolyses cAMP in the phagocyte, decreasing the levels of this important second messenger, which regulates essential cell signaling events that determine many cell functions [77], [78]. Previous studies have shown that increased levels of cAMP modulate immune cell functions, including the respiratory burst, chemotaxis, phagocytosis and phagosome maturation [79], [80]. Consistent with these findings, treatment of *Mtb* infected rabbits with CC-3052 significantly reduced the steady state mRNA levels of TNF-α and other pro-inflammatory, Th1 and Th2 cytokines, chemokines and their receptors, apoptosis-associated genes, surface receptors, and signaling molecules in macrophages. TNF-α plays a central role in the host innate immune response and specifically in the activation of macrophages [81]. This cytokine is primarily regulated via TLR signaling through activation of the NFκB pathway [82], [83]. Thus, the reduced level of expression of both the TNF-α and NFκB genes in the lungs of *Mtb* infected rabbits treated with CC-3052 suggests a suboptimal activation of macrophages in the lesions of these animals [84].

Suboptimal activation of macrophages has been shown to affect the cellular organization in the lung granulomas of *Mtb* infected animals [49]. Briefly, in *Mtb* infected animals, there were no significant differences either in the total number of subpleural lung granulomas or in the area of lung involved, between the untreated and CC-3052 treated rabbits up to 12 weeks post-infection. However, the granulomas in the lungs of CC-3052 treated rabbits appeared less necrotic, less fibrotic and more cellular compared to the untreated rabbits, most strikingly at 12 weeks post-infection (8 weeks of CC-3052 treatment). In mice, CC-3052 treatment affected the distribution of T cells in the lung lesions without affecting their total numbers. When *Mtb* infected animals (both mice and rabbits) were treated with INH plus CC-3052, a significant reduction in the size and number of lung granulomas was noted, compared to INH treatment alone. Whether these morphologic changes affected drug penetration and/or exposure of the bacilli to INH in the granulomas is as yet unknown and will be investigated in the future.

Macrophage activation during infection is associated with phagosome maturation, as indicated by the acquisition of RAB7 and LAMP2 on the organelle surface [59], [85]. The reduced expression of *RAB7* and *LAMP2* in response to CC-3052 treatment suggests that there may indeed have been impaired or suboptimal macrophage phagosome maturation in the immune modulated rabbits. In addition, macrophage activation during *Mtb* infection is characterized by the production of a number of pro-inflammatory cytokines, chemokines, growth factors and their receptors. Increased TNF-α production by activated macrophages leads to increased expression of apoptotic genes, such as *FAS*, and reduced expression of anti-apoptotic gene expression, such as *BCL-X* [60]. The reduced expression of *FAS* and increased expression of *BCL-X* observed in rabbits treated with CC-3052 suggests reduced cell apoptosis during treatment with the PDE4i. Overall, the pattern of change in host gene expression in the lungs of *Mtb* infected rabbits treated with CC-3052, compared to untreated rabbits, was similar to that seen in resting macrophages cultured in vitro, where expression of the genes encoding for proinflammatory cytokines, chemokines, and their receptors was relatively low [86]–[88].

The modulation of host innate immunity by CC-3052 treatment appeared to be sensed by the infecting intracellular *Mtb*, resulting in corresponding changes in the expression of many bacterial genes. How would changes in macrophage activation be expected to affect the physiology of the infecting bacilli? First, the observation that expression of many of the *Mtb* stress response genes induced during infection was significantly reduced by CC-3052 treatment supports our hypothesis that *Mtb* adapted to perturbations in the host environment [14], [23], [73], [89]–[92]. The metabolic shift-down of selected *Mtb* genes during non-replicating persistence is mediated by the activation of *devR*, a global transcriptional regulator, that up-regulates at least 53 *Mtb* genes involved in the bacterial adaptation to dormancy [93], [62]. Among these are the *Mtb* stress response genes, *narX* and *narK2*, which are induced when the infecting bacilli enter into dormancy [65], [94], [95]. In the present study, CC-3052 treatment reduced the expression of *devR*, *narX*, and *narK2* in the infected rabbit lungs, suggesting that the bacilli sensed the reduction in environmental pressure within suboptimally activated macrophages. Treatment with the PDE4i also lead to reduced expression of the transcriptional regulator *relA*, which is essential for *Mtb* survival under stress conditions, as well as for infection, persistence and dissemination in mice and guinea pigs [96], [97]. Consistent with our findings, loss of *relA* expression in *Mtb* has been shown to be associated with reduced lung inflammation and pathology in infected guinea pigs [97]. Similarly, mycobacterial genes encoding superoxide dismutases (*sodA* and *sodC*), heat shock protein (*hspX*) and stress response sigma factors (*sigF* and *sigH*) have been shown to be associated with adaptation of the bacilli to a stressful environment, such as hypoxia, within activated macrophages [98]–[100]. The mRNA levels of these mycobacterial genes (*sodA*, *sodC, sigF* and *sigH*) were down-regulated during CC-3052 treatment, consistent with adaptation of the bacilli to a less stressful environment. Differential regulation of the sigma factors ensures the regulation of a distinct set of *Mtb* genes essential for survival under various environmental conditions such as availability of nutrients, oxygen status, and the presence of antibacterial molecules. [101]. Moreover, the up-regulation of a set of *Mtb* genes involved in protein synthesis, cell wall/lipid metabolism, iron metabolism and ESX-3/secretion pathways in response to CC-3052 treatment was similar to the pattern observed for intracellular *Mtb* inside resting macrophages [64]. Interestingly, cycloproponation of *Mtb* cell wall mycolates by pcaA has been shown to be essential for the immune recognition of *Mtb* through trehalose dimycolate (TDM), an inflammatory glycolipid unique for pathogenic *Mtb* [102], [103]. Furthermore, lack of mycobacterial *pcaA* gene expression was shown to reduce the level of TNF-α and other proinflammatory cytokines in *Mtb* infected murine bone marrow macrophages [102]. Taken together, the results of our study support the existence of mutually regulated host-pathogen interactions during *Mtb* infection. Both the infected host cells and the infecting bacilli appear to fine-tune their gene expression during adaptation to a state of intracellular infection. When the natural infection-induced macrophage activation was dampened in CC-3052 treated animals, as evidenced by the reduced expression of innate immune response genes, the bacilli sensed the change and reset their metabolism by altering their own gene expression.

Remarkably, changes in the gene expression profile of the bacilli, albeit limited in magnitude, appeared to affect the ability of INH to kill the bacteria. It is conceivable that, when the transcriptional regulation of *Mtb* genes, such as those governed by *devR*, *relA* and alternate sigma factors, associated with the dormant/non-replicative state is altered, the bacilli are forced out of the non-replicating, persistent drug tolerant state [104], [105]. Thus, a change in the microenvironment wherein the infecting *Mtb* reside would significantly influence the bacterial vulnerability to antibiotic killing. Consistent with this hypothesis, we observed improved bacterial killing in the lungs of *Mtb* infected rabbits treated with INH plus CC-3052 compared to INH alone. Increased expression of the INH-induced *Mtb* genes following 4 weeks of INH treatment was an indication of bacterial exposure to INH, despite the minimal bactericidal activity observed at this time point. In contrast, 4 weeks of INH treatment in the presence of CC-3052 led to reduced levels of expression of these genes. Though the exact mechanism of down-regulation of INH-responsive *Mtb* gene expression during CC-3052 co-treatment is currently unknown, this observation may be linked to the improved killing of *Mtb* by INH plus CC-3052. The results of a number of previously published studies may offer alternative non-exclusive explanations for our findings. INH is a pro-drug that is activated by the bacterial catalase-peroxidase (KatG) to form functional adducts with NAD, which inhibit InhA, an important enzyme involved in the cell wall synthesis of *Mtb* [106]–[107]. In addition to INH activation, KatG also possesses peroxynitritase and NADH oxidase activities that are vital for bacterial defense against oxidative stress [108]–[109]. Thus, as we have observed in the CC-3052 treated rabbits, under reduced oxidative stress conditions, *katG* expression would be expected not to be up-regulated. Furthermore, induction of mycobacterial efflux pumps and their regulators, in response to intracellular stress conditions during macrophage infection of *Mtb,* has been reported [110]–[112]. Recently, increased expression of an efflux pump was reported to be associated with tolerance of *M. marinum* to INH in a zebrafish model of mycobacterial infection [113]. Moreover, up-regulation of *efpA*, encoding for an efflux pump in *Mtb*, has been reported in MDR-TB [114]. In the present study, the reduced expression of *efpA* in CC-3052 treated rabbits may contribute to the improved INH-mediated killing observed. Recently, by screening a chemical library of 300 compounds for their inhibitory activity against enoyl reductase (InhA), Vilcheze et.al identified two potential targets, CD39 and CD117, that exhibited significantly increased *Mtb* killing when combined with INH, compared to treatment by either of the compounds separately or INH alone [115]. This finding suggests that chemicals that reduce/inhibit InhA could facilitate improved *Mtb* killing when co-administered with INH. A similar mechanism may be operative in our studies where inhibition of expression of *inhA* by CC-3052 treatment of the rabbits was associated with an increase in INH-killing of *Mtb*.

Studies from our group and others have shown that generalized immune suppression by anti-TNF-α antibody treatment in the mouse model of pulmonary TB compromised the ability of the animals to control bacillary growth, and exacerbated the pathology in the lungs [84], [116], [117]. Unlike anti-TNF-α antibody treatment and similar to our data in the mouse, CC-3052-mediated PDE4 inhibition had no effect on bacillary growth in the lungs of infected rabbits [49]. The use of PDE4 inhibition as a means to reduce, but not abolish, TNF-α levels has the selective advantage of targeting the level of production of the cytokine in monocytes and macrophages. Thus, T cells and other cells of the immune response would retain their ability to produce TNF-α, facilitating control of *Mtb* infection in the lungs. In contrast, complete neutralization of TNF-α production by treatment with an antibody against the cytokine would be expected to render the animals immune suppressed, thereby leading to uncontrolled bacillary growth and death of the animals [118]–[121], [94]. In addition, in vitro culture of *Mtb* in the presence of CC-3052 had no effect on the growth of the bacilli, confirming that the PDE4i had no direct effect on bacillary growth or killing, but rather affected the bacilli in vivo via modifying the host cell (Figure S2). Thus, dampening innate immunity without interfering with the development of the acquired immune response was associated with changes in the metabolic activity of *Mtb* but not with loss of control of bacillary growth. Previous reports have demonstrated the usefulness of TNF-α inhibition by CC-3052 treatment, both in acute and chronic HIV-1 infection in vitro [122], [123]. In addition, the safe administration of several PDE4i has been demonstrated in clinical trials for the treatment of other inflammatory diseases, including asthma and chronic obstructive pulmonary disease [124]–[126]. An adjunctive therapeutic approach, such as the one described in this study, could be used to treat humans with TB, providing a means for shortening treatment and improving clinical outcome in patients with active disease. The absence of significant immune suppression or other toxicities supports the idea that such an approach may safely contribute to improved treatment of TB.

Taken together, our observations support the hypothesis that changes in the physiology of the bacteria, in response to changes in the host immune response can alter the susceptibility of the bacteria to antimicrobial agents. This is a novel strategy to combat *Mtb* infection, facilitating the use of the existing drugs more efficiently. As different anti-TB drugs target both overlapping and diverse bacterial functions, it is important to evaluate immune modulation during other single as well as multidrug treatments. Currently we are testing this approach by combining CC-3052 with other anti-TB drugs in our rabbit model of pulmonary TB.

## Materials and Methods

### Ethics statement

All rabbit experiments were performed according to the procedures and policies of the Animal Welfare Act guidelines for housing and care of laboratory animals and conducted in accordance with Public Health Service Policy Institutional regulations. Animal ethics approval for rabbit aerosol infection with *Mtb*, treatment, post-treatment care, euthanasia and necropsy was obtained from the Institutional Animal Care and Use Committee (IACUC) and Institutional Biosafety Committee (IBC) of the Public Health Research Institute (PHRI) at University of Medicine and Dentistry of New Jersey (UMDNJ) (IACUC approval numbers 070,124 and 125). All procedures with the infected animals/tissues were performed in a Biosafety level three (BSL-3) containment facilities according to the approved protocols.

### Mycobacterial culture conditions and chemicals

*Mycobacterium tuberculosis* HN878 (*Mtb*) (a gift from Dr. Musser, TX, USA), a member of Beijing strains, was grown to mid-log phase (OD_600_ = 0.6–0.7) in Middlebrook 7H9 liquid media (Difco, MI, USA) supplemented with 0.5% glycerol, 10% OADC enrichment (oleic acid, albumin, dextrose and catalase; BD Biosciences, MD, USA) and 0.25% Tween 80 at 37°C and 5% CO_2_ as static culture. The culture was gently mixed once a day for proper aeration. Serial dilutions of the culture were enumerated for the number of colony forming units (CFU) by plating on 7H10 agar plates (Difco, MI, USA) followed by incubation of the plates at 37°C and 5% CO_2_ for 4–5 weeks. All the stock cultures were stored as aliquots at −80°C until use. A vial of frozen stock culture was thawed at 37°C, sonicated three times in 5-seconds pulses on ice to disrupt bacterial clumps, diluted to 5×10^6^ CFU/ml in sterile saline with 0.05% Tween 80 and used for rabbit aerosol infection [127]. The PDE4 inhibitor used in this study, CC-3052, was a kind gift from Celgene Corporation (Celgene Corporation, NJ, USA). All other chemicals were purchased from Sigma (St. Louis, MI, USA) unless otherwise mentioned.

To study the effect of CC-3052 and/or INH on *Mtb* growth in vitro, bacterial pellets from mid-log culture were washed with sterile PBS and resuspended in complete 7H9 media. About 1×10^6^ CFU/ml of the bacteria in complete 7H9 media was seeded in to 24 well plates and various concentrations of INH (0.0125 µg or 0.2 µg per ml), CC-3052 (4 or 40 or 200 µM) or a combination of both (CC-3052+INH) were added every day up to 4 days. The number of bacterial CFU was enumerated for the initial inoculum and after every 24 hours of culture by plating serially diluted bacterial suspensions (treated and untreated) on 7H10 agar plates followed by incubation of the plates at 37°C and 5% CO_2_ for 4–5 weeks.

### Rabbit aerosol infections

A total of 84 male and female, specific pathogen-free, New Zealand White rabbits of approximately 2.5 kg in weight, purchased from Millbrook Farms (Millbrook Farms, MA,USA) were used for all the experiments reported in this study. The animals were acclimatized for a week after arrival at the PHRI Research Animal Facility before exposure to aerosol challenge. Groups of 6 rabbits (per round of infection) were infected with *Mtb*, using a nose-only aerosol exposure system (CH Technologies, Inc., NJ, USA) as described earlier [127]. Twelve to twenty-four rabbits were infected for each experiment. After 3 hours post-exposure, one group of infected animals were sedated with a combination of Ketamine and Xylazine and euthanized by Euthasol and necropsy performed to determine the bacillary load (T = 0) implanted in the lungs as described earlier [128]. For all the experiments, the bacterial inoculum, exposure time and conditions were standardized so as to implant approximately 3.2 log_10_ CFU in the lungs at T = 0. Infected rabbits were housed individually in a BSL-3 facility, with an unrestricted food and water supply. At defined time points (T = 4, 8 and 12 weeks post-infection), groups of 2–4 rabbits, per treatment and time point, were euthanized and the lung, liver and spleen aseptically removed and portions of each organs used for CFU enumeration and isolation of host and bacterial RNA. The tissue segments for RNA isolation were frozen immediately in liquid nitrogen and stored at −80°C.

### Treatment of *Mtb* infected rabbits

The *Mtb* infected rabbits were classified into the following treatment groups: **1. CC-3052 Treatment.** The immune modulatory drug used in this study, CC-3052, is an analog of thalidomide. Solutions of CC-3052 were prepared, freshly every day, in sterile water and administered through gavage at a dose of 25 mg/kg body weight using a flexible rubber feeding tube 5 days per week. Treatment with CC-3052 started at 4 weeks post-infection and continued until 12 weeks after infection (8 weeks of treatment). **2. Isoniazid (INH) Chemotherapy.** Freshly prepared INH, at 25 or 50 mg/kg body weight was administered to rabbits through oral administration using a flexible rubber feeding tube 5 days a week. The INH treatment was started at 4 weeks post-infection and continued until 12 weeks after *Mtb* infection (8 weeks of treatment). **3. INH plus CC-3052 Combination Therapy.** Rabbits were treated with a combination of INH (50 mg/kg body weight) plus CC-3052 (25 mg/kg body weight) using a flexible rubber feeding tube 5 days per week. Treatment was initiated concurrently at 4 weeks post-infection and continued until 12 weeks after *Mtb* infection (8 weeks of treatment).

All three treatments were carried out in parallel. One group each of uninfected and infected but untreated rabbits served as controls for each time point of the experiment.

### Bacillary load estimation

Bacterial loads in the lungs, liver and spleens of the *Mtb* infected rabbits were enumerated by CFU assay in respective organs at each time point of the experiment. Briefly, random portions of lungs (about 1/3 of the entire lung), liver (about 1/10) and spleen (about half) tissue homogenates in sterile saline were serially diluted and plated onto Middlebrook 7H11 agar plates (Difco, MI, USA) with or without INH supplement (0.2 µg/ml). The plates were incubated at 37°C for 4 to 5 weeks. Colonies were counted, and results were calculated for total numbers of CFU in the whole organ.

### Host and mycobacterial total RNA isolation

Total RNA of the host and *Mtb* were isolated from the lung tissues of all four groups of rabbit (infected, infected and CC-3052-, INH- or INH+CC-3052- treated and uninfected) at each time point of necropsy (T = 0, 4, 8 and 12 weeks post-infection). Tissue sections from each rabbit were processed separately for host and bacterial RNA extraction. To prepare host RNA, lung tissue slices were homogenized in 10 volumes (wt/vol) of TRIzol (Invitrogen, CA, USA) using a PolyTron homogenizer (Kinematica, Lucerne, Switzerland), and extracted with 0.3 vol (vol/vol) of chloroform. The mixture was centrifuged at 13,000 rpm for 20 minutes at 4°C and the cleared supernatant was added to an equal volume of precipitation solution (Macherey-Nagel, GmbH) and eluted through the NucleoSpin kit as per the manufacturer's protocol (Macherey-Nagel, GmbH). For bacterial total RNA isolation from infected rabbit lungs, a modified differential lysis method was used [129]. Briefly, the tissue sections were homogenized in 10 volumes of sterile 0.01% SDS solution, followed by centrifugation at 13,000 rpm for 20 minutes at 15°C to pellet the bacteria. The bacterial pellet was resuspended in 10 volume of TRIzol (wt/vol) and the mixture was subjected to bead beating with Ribo-lyser (MP Biosciences, OK, USA) for 2 minutes in 30-seconds pulses with 1-minute ice-incubation in between the pulses. The lysate was extracted with equal volume of chloroform (vol/vol) followed by centrifugation and the cleared supernatant was eluted using NucleoSpin kit as per the manufacturer's protocol (Macherey-Nagel, GmbH). Both host and bacterial RNA were subjected to DNase I digestion before final purification through RNeasy mini kit (Qiagen, MD, USA). The quantity and quality of the total RNA was estimated by NanoDrop (NanoDrop Products, DE, USA) and agarose gel electrophoresis as reported elsewhere [130].

### Rabbit microarray analysis

The rabbit microarray slides and the recommended reagents were purchased from Agilent Technologies (Agilent Technologies, CA, USA). According to the manufacturer, each rabbit microarray slide contains quadruplicates of 43,604 probes (4 arrays per slide) of rabbit genome, derived from public domain databases, corresponding to 43,604 open reading frames (ORFs). The total lung RNA from individual rabbits (2–4 animals per time point per group) was processed separately for each microarray experiment. Dye-swap was done for every group, to avoid dye bias during cDNA labeling, hybridization and post-hybridization procedures. The conditions for cDNA synthesis, labeling and hybridization were according to the standard operating protocol of the Center for Advanced Genomics of PHRI (refer <http://www.cag.icph.org/downloads_page.htm>). For the 4 weeks time point, the gene expression values of the *Mtb* infected rabbits were calculated relative to uninfected rabbits (Infected vs. Uninfected) and for all other time points, the gene expression values of CC-3052 treated rabbits were calibrated against untreated (but *Mtb* infected) rabbits at the same time point (CC-3052 treated vs. untreated). The slides were scanned and analyzed by Agilent Scanner and Feature Extraction Software (Agilent Technologies, CA, USA) and the acquired data was loaded into Partek Genomics Suite (PARTEK, MO, USA) for further analysis after appropriate statistical analysis to adjust for the errors. A 2.0 fold difference in expression value and a *p* value of less than 0.05 was set as cut-off to select differentially expressed genes between various comparison groups. The selected list of differentially expressed rabbit genes from PARTEK Genomics Suite was further analyzed for functional classification including derivation of networks and pathways using Ingenuity Pathway Analysis (IPA) software (Ingenuity Pathway Analysis, CA, USA). Since the information on the functional characterization of rabbit genes is currently unavailable with any of the commercial software including IPA, we used the gene information from human, mouse and rat database, from various microarray platforms, available in IPA. We searched the IPA knowledge base for conserved homologous of rabbit genes that perform similar functions and mostly share common pathways, across the 3 different species (mouse, rat and human). The filtered list of rabbit genes, containing “pathway eligible genes” was classified according to their functions in specific pathways and used to construct corresponding networks in IPA. The complete list of rabbit genes differentially expressed by CC-3052 treatment has been submitted to the GEO database (GSE27992).

### Quantitative Real Time PCR Analysis (qRT-PCR)

Total RNA of the host and *Mtb*, isolated from the uninfected/infected rabbit lungs were subjected to cDNA synthesis using Sprint RT Complete kit as described by the manufacturer (Clontech, CA, USA). The cDNA was amplified with gene specific primers and SYBR green mix as per the manufacturer's instructions (Clontech, CA, USA) in a MxPro4000 real time PCR machine (Stratagene, CA, USA). The SYBR green qRT-PCR mix also contains ROX as an internal reference dye. The primers for specific rabbit and *Mtb* genes were designed using Clone Manager Suite (Sci-Ed software, NC, USA). The DNA (for *Mtb*) and mRNA (for rabbit) sequences of specific *Mtb* and rabbit genes were obtained from Tuberculist (for *Mtb* genes) or GenBank (for rabbit genes) data base. The DNA sequences of the primers used for qRT-PCR can be found in Table S1 and S2. The threshold cycle (Ct) for each amplified target gene was calculated using MxPro4000 software (Stratagene, CA, USA). Uniform baseline fluorescence was set for all the genes in each experiment and across different experiments. The *Mtb* gene for 16S rRNA and the transcripts for rabbit GAPDH gene were used to normalize the Ct values of the target genes. Fold change was calculated using the formula 2^−ΔΔCt^ and represented either as absolute transcript levels or as relative expression after normalization to uninfected or untreated groups. The experiments were repeated at least 3 times with RNA samples from two to four animals per group per time point per treatment group.

### Isoniazid measurement in rabbit plasma

The concentration of INH in the plasma of *Mtb* infected and treated rabbits were measured using Sciex API4000 (Applied Biosystems, CA, USA) mass spectrometer coupled to Symbiosis Pharma HPLC system (Spark Holland B.V, The Netherlands). Briefly, one part of the plasma or homogenized lung tissue was extracted with nine parts (vol/vol) of acetonitrile containing 0.2% acetic acid. Ten microliter of the supernatant was injected into a Phenomenex Gemini C6-Phenyl column (4.6×150 mm) (Phenomenex, CA, USA) and eluted with a gradient of mobile phase A (0.2% acetic acid in deionized water) and B (0.2% acetic acid in methanol) at a flow rate of 1 ml/min. Presence of the INH analytes and quantification of drug levels were performed by monitoring multiple reactions of parent/daughter transitions in electro-spray positive ionization mode. The test samples, standards and quality control samples were spiked with Warfarin, which was used as an internal standard. The calibration curve was designed to cover the expected concentration range (10.2 ng/ml to 10.4 µg/ml) of samples delivered and was derived from standard solutions of INH. The data obtained was processed using Analyst software v 1.4.2 (Applied Biosystems, CA, USA) and regression for pharmacokinetic parameters was performed as non-compartmental analysis using WinNonLin 5.0 (Pharsight, CA, USA).

### Statistical analysis

The independent Student *t*-test or the Mann-Whitney test for nonparametric independent data was used for analysis (SPSS software). *p*≤0.05 was considered significant for all the experiments.

## Supporting Information

Figure S1 **Effect of CC-3052 on the expression of dormancy-related genes of** ***Mtb.*** Log phase *Mtb* culture was exposed to 4uM of CC-3052 for 24 hours and total bacterial RNA was isolated. The untreated culture was added with equimolar amount of the vehicle (DMSO) and processed similarly for RNA isolation. Results shown are mean ± standard deviation from at least 2 experiments done in duplicate. No statistically significant difference in the dormancy related *Mtb* gene expression was observed between untreated and CC-3052 treated bacteria.
(TIF)

Figure S2 **The effect of CC-3052 and INH on the growth of** ***Mtb*** **in vitro.** The number of *Mtb* CFU (in log_10_ scale) during treatment with various concentrations of CC-3052 (A) or INH (B) or both (C) up to 4 days in Middlebrook 7H9 liquid media. The concentrations of CC-3052 are in micromoles and of INH are in micrograms; UT-untreated. Results shown are mean ± standard deviation from at least 2 experiments done in triplicate (total of at least 6 data points).
(TIF)

Table S1 **List of** ***Mtb*** **primers used in qRT-PCR experiments.** The DNA sequences of listed *Mtb* genes were obtained from Tuberculist (http://genolist.pasteur.fr/TubercuList/) using the gene names to search for the sequence.
(XLS)

Table S2 **List of rabbit primers used in qRT-PCR experiments.** The DNA sequences of listed rabbit genes were obtained from GenBank (http://www.ncbi.nlm.nih.gov/genbank/) using the gene names to search for the sequence.
(XLS)
