# Supplementary figures and images for: Phosphodiesterase-4 Inhibition Alters Gene Expression and Improves Isoniazid – Mediated Clearance of Mycobacterium tuberculosis in Rabbit Lungs
Source: PLoS Pathog. 2011 Sep 15;7(9):e1002262. doi: 10.1371/journal.ppat.1002262 (PMC3174258; doi:10.1371/journal.ppat.1002262)

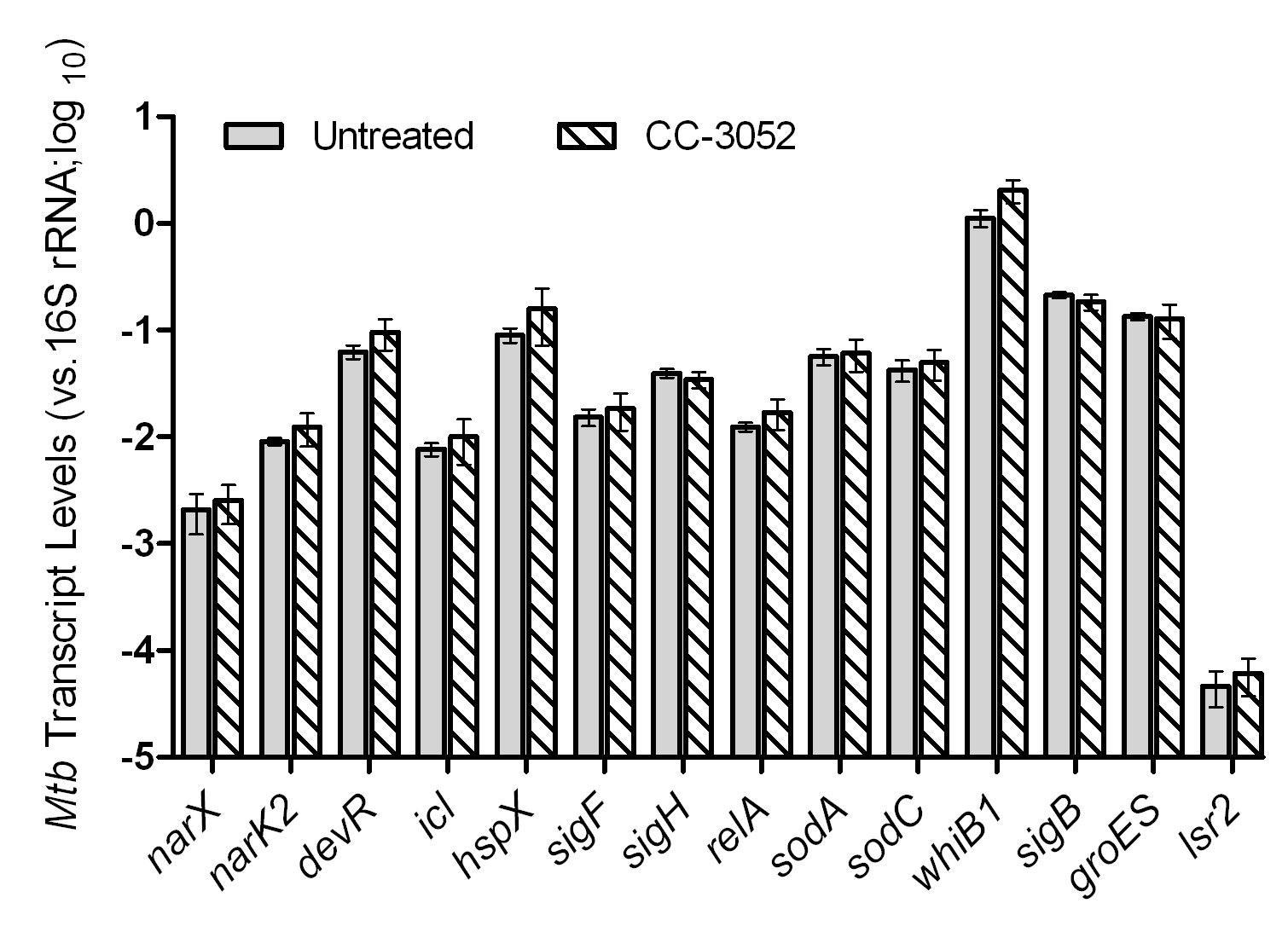

Supplement: Figure S1 — Effect of CC-3052 on the expression of dormancy-related genes of Mtb. Log phase Mtb culture was exposed to 4uM of CC-3052 for 24 hours and total bacterial RNA was isolated. The untreated culture was added with equimolar amount of the vehicle (DMSO) and processed similarly for RNA isolation. Results shown are mean ± standard deviation from at least 2 experiments done in duplicate. No statistically significant difference in the dormancy related Mtb gene expression was observed between untreated and CC-3052 treated bacteria. (TIF) [file ppat.1002262.s001.tif]

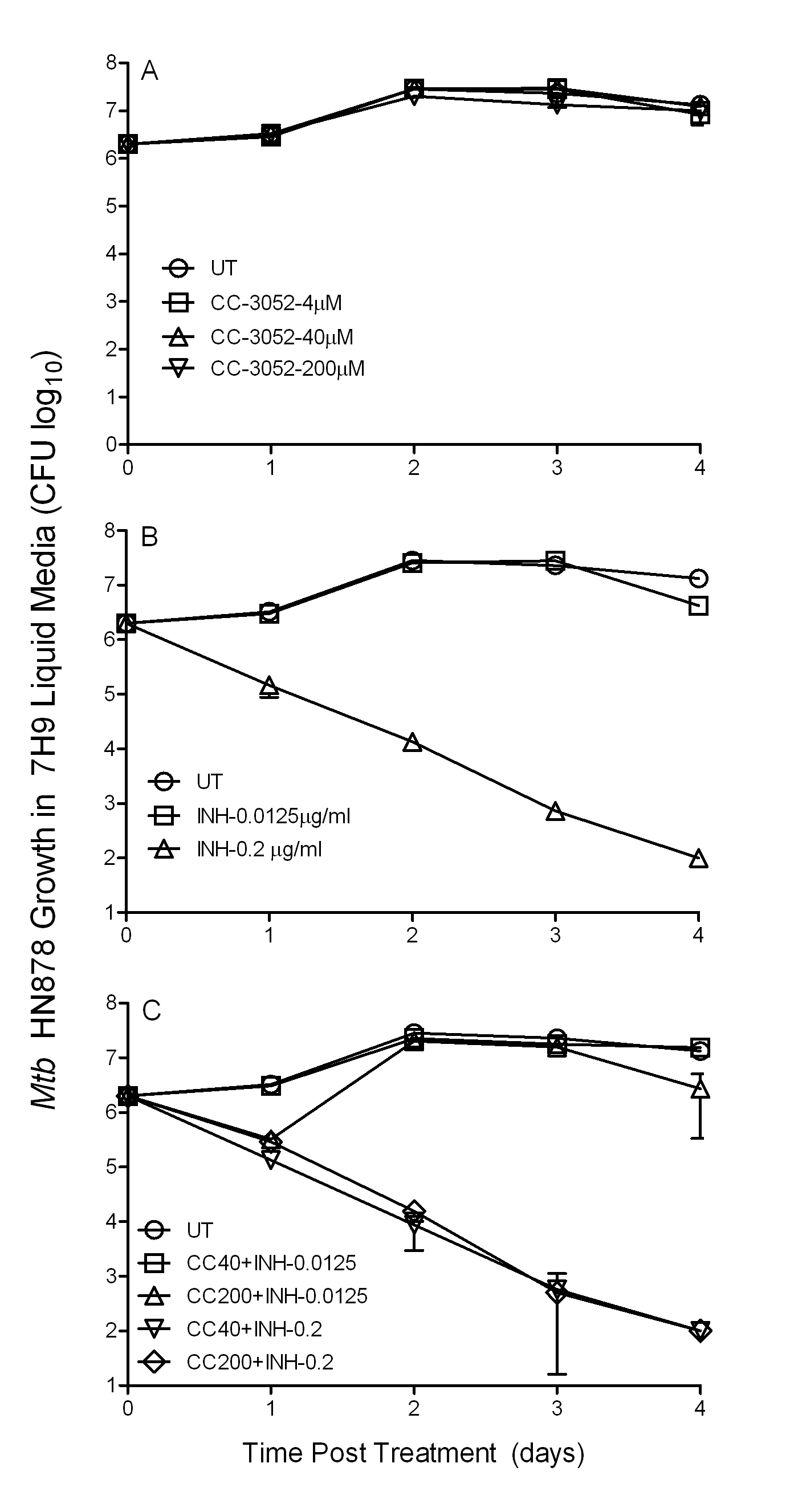

Supplement: Figure S2 — The effect of CC-3052 and INH on the growth of Mtb in vitro. The number of Mtb CFU (in log10 scale) during treatment with various concentrations of CC-3052 (A) or INH (B) or both (C) up to 4 days in Middlebrook 7H9 liquid media. The concentrations of CC-3052 are in micromoles and of INH are in micrograms; UT-untreated. Results shown are mean ± standard deviation from at least 2 experiments done in triplicate (total of at least 6 data points). (TIF) [file ppat.1002262.s002.tif]
